# Waiting Narratives of Lung Transplant Candidates

**DOI:** 10.1155/2013/794698

**Published:** 2013-02-12

**Authors:** Maria T. Yelle, Patricia E. Stevens, Dorothy M. Lanuza

**Affiliations:** ^1^School of Nursing, Edgewood College, 1000 Edgewood, College Drive, Madison, WI 53711-1997, USA; ^2^College of Nursing, University of Wisconsin-Milwaukee, P.O. Box 413, Milwaukee, WI 53201-0413, USA; ^3^School of Nursing, University of Wisconsin-Madison, F6/1866 CSC, 600 Highland Ave, Madison, WI 53792-2455, USA

## Abstract

Before 2005, time accrued on the lung transplant waiting list counted towards who was next in line for a donor lung. Then in 2005 the lung allocation scoring system was implemented, which meant the higher the illness severity scores, the higher the priority on the transplant list. Little is known of the lung transplant candidates who were listed before 2005 and were caught in the transition when the lung allocation scoring system was implemented. A narrative analysis was conducted to explore the illness narratives of seven lung transplant candidates between 2006 and 2007. Arthur Kleinman's concept of illness narratives was used as a conceptual framework for this study to give voice to the illness narratives of lung transplant candidates. Results of this study illustrate that lung transplant candidates expressed a need to tell their personal story of waiting and to be heard. Recommendation from this study calls for healthcare providers to create the time to enable illness narratives of the suffering of waiting to be told. Narrative skills of listening to stories of emotional suffering would enhance how healthcare providers could attend to patients' stories and hear what is most meaningful in their lives.

## 1. Introduction

Lung transplant candidates suffer from chronic end-stage respiratory disease such as chronic obstructive lung diseases, cystic fibrosis, idiopathic pulmonary fibrosis, and pulmonary hypertension. Lung transplant candidates were referred by their physician to be considered as a candidate for an organ transplant because they are no longer responding to the maximum level of optimal medical therapy for their condition. Candidates must undergo a series of tests that thoroughly examine their physical status, mental status and social support networks. If the individual is accepted, they are added to a national patient waiting list for lung organ transplant called The Organ Procurement and Transplantation Network (OPTN), which is the national organ procurement, donation, and transplantation system. OPTN works alongside with the United Network for Organ Sharing (UNOS) which is a nonprofit organization that maintains a centralized data base that links all organ procurement organizations and transplant centers in the nation [1, 2]. 

In May of 2005 the OPTN and UNOS changed the way they allocated organs to lung transplant candidates. The Lung Allocation Scoring System was implemented, which was a new method that utilizes a scoring system that will determine the candidates position on the transplant waiting list [3]. The lung allocation system uses medical information such as lab values, test results, and disease diagnosis that are used to calculate a score from 0 to100 for each lung transplant candidate. This lung allocation score represents an estimate of how severe their condition is and their ranking on the waiting list. Before May 2005, the scoring system was based on time accrued on the waiting list, with the approximate wait time being two years [3]. Therefore, the longer you were on the waiting list the closer you were to being the next to be transplanted.

Since the lung allocation scoring system was implemented, the lung transplant candidates' medical information and lung allocation scores are updated into the system every six months. The higher the scores reflect the higher urgency for transplant, and those candidates are considered as priority to be transplanted. The lung allocation scores can fluctuate over time depending on the candidates' condition. 

When a deceased organ donor is identified, a transplant coordinator from an organ procurement organization accesses the UNOS database. The computer then generates a ranked list of candidates for each available organ in ranked order according to OPTN organ allocation policies. The factors considered are as follows: is there a match between donor and lung transplant candidate, such as tissue match, blood type, immune status, and geographic distance between the potential lung transplant recipient and the donor (UNOS). Lung transplant candidates must agree to be ready for the call at any time when a donor match is identified. 

Since the changes were implemented, little is known of the individuals who were affected by the changes and were no longer a priority for transplant based on the new rules. Also a paucity of research has explored barriers to express the emotional toll of waiting. This study is a secondary analysis of Lanuza et al.'s (unpublished) parent study, Symptom Management and Self-Care Monitoring in Lung Transplant Candidates. The purpose of Lanuza et al.'s parent study was to test the feasibility of a pretransplant, patient-centered educational intervention using the representational approach. The representational approach intervention asks the participant to describe what they identify as their symptom, what they think is the cause, timeline, consequence/impact, and control or cure from their point of view [4, 5]. The purpose of this secondary analysis was to investigate the illness narratives of lung transplant candidates.

## 2. Background

Lung transplant candidates experience high levels of anxiety, depression, and mood disorders during the waiting period [6]. Social desirability was found to be a factor in self-report measures of depression, anxiety, and negative mood scores [7]. Lung transplant candidates have lower quality of life scores as compared to posttransplant recipients [8–14].

Three patient-centered intervention studies, which focused on psychological distress and coping during the waiting period, showed that interventions improve psychological measures of depression and anxiety [15–17]. Lung transplant candidates use adaptive problem and emotion focused coping strategies [18]. Avoidant coping has been associated with low quality of life measures [19]. However, little is known from the lung transplant candidate's point of view.

Four qualitative studies examined the experiences of lung transplant candidates before changes to the lung allocation system [20–23]. Moloney et al. conducted a study to identify the lung transplant candidate's informational needs and role of support person to make an informed decision [20].

Macdonald conducted a content analysis guided by Lazarus and Folkman's theory of coping [21]. This content analysis explored the lived experience of cystic fibrosis sufferers and their caregivers coping with the rigors of chronic illness as they wait for transplant. The sample consisted of eight participants with cystic fibrosis (*N* = 4 waiting for transplant and *N* = 4 posttransplant) and 5 of their caregivers. The mean age of participants was 28.8 years old with a standard deviation of 8.45. Four major themes emerged from the data: (1) displacement, (2) disorder, (3) need for support, and (4) life in limbo. Findings of the theme “displacement” centered on reactions that displaced the candidates' “status quo” as people who live with the chronic condition of cystic fibrosis throughout their lives. They expressed shock that they were referred to as lung transplant candidates because this was a normal way for them to live. The theme “disorder” centered on the uncertainty during the evaluation process. The third theme, “need for support,” explored the importance of having a mentor who has been through the waiting period, especially during times when the candidate was turned down for a transplant because the donor organ was not a match. The fourth theme, “life in limbo,” is named for the period that participants described as “buying time waiting for their beeper to go off.” Lung transplant candidates have to be available at any time to be ready for the call for transplant.

Naef and Bournes conducted phenomenological research using Parse's method of human becoming in order to investigate the experiences of lung transplant candidates during the waiting period. Eleven participants with various advanced lung diseases were interviewed (eight women and three men). Participants were interviewed one time and an in-depth analysis of the experiences of each participant was conducted using the Parse approach. The findings revealed that the lived experience of waiting is an immensely difficult and agonizing experience of persistently expecting a prized opportunity, finding strength in being with other people, and engaging in diverse activities to stay occupied [22]. 

Yorke and Cameron-Traub explored heart and lung transplant candidates' perceived needs for care from their nurses at the transplant center [23]. This study employed a descriptive, longitudinal, repeated-measures thematic analysis. The authors conducted interviews on three separate occasions, one to two weeks apart. The research questions in the first interview were “How have the nurses assisted you during the waiting period? Tell me what has been helpful or not helpful? What is important to you in having quality nursing care?” In the second interview, the questions were “How have the nurses supported you during the waiting time? What do you find comforting?” The third interview asked “Has anything changed since the last interview? Tell me what the nurses have done for you?” Overall, Yorke and Cameron-Traub found five themes in transplant candidates' perceived needs, which are dominated by the patients' psychological needs from their nurses: (1) importance of information giving, (2) maintenance of regular contact with nurses, (3) familiarity, (4) positive thinking, and (5) compassionate manner [23]. 

A gap exists in knowledge about lung transplant candidates' experiences of waiting since the changes in the lung allocation scoring system. Little is known of how the implementation of the lung allocation scoring system influences the emotional toll of waiting for a transplant. Therefore, for this secondary analysis, a qualitative approach using narrative analysis was conducted to investigate the illness narratives of the lung transplant candidates. 

### 2.1. Illness Narratives

Illness narratives are a response to biomedicine's focus on the disease narrative voice and the consequential neglect of the patient's embodied experience of illness [24–27]. An illness narrative is “the story the patient tells, and significant others retell to give coherence to the distinctive events and long-term course of suffering. The plot lines, core metaphors and rhetorical devices that structure the illness narrative are drawn from cultural and personal models for arranging the experiences in meaningful ways and for effectively communicating those meanings” (pg. 49) [28]. 

Kleinman et al. introduce the explanatory model, a clinical strategy that centers on the process of communication between the patient and the healthcare provider [29]. Explanatory models open up for clinicians the complexity of human communication [30]. Kleinman states “… illness has particular meanings for practitioners who listen to patient's accounts of illness in light of their own special interests (therapeutic, scientific, professional, financial, personal)” (pg. 52) [28]. Therefore, healthcare providers must be cognizant of their own explanatory models in order to be able to engage with the patient's explanatory models, which he describes as “illness narratives.” Arthur Frank, a medical sociologist, wrote *The Wounded Storyteller* where Frank provides great insight into how patients surrender their own personal narrative to the physician's medical narrative [31]. 

There has been a long history of using narrative to study the patient's experience of illness. According to Hydén illness narrative research has been a project shared by disciplines in the humanities, sociology, anthropology, medicine, and bioethics [32]. These studies are interested in understanding human suffering caused by the body's deteriorations, whether it be through aging, disability, or chronic illness. Chronic illness alters the relationship between the patient's body, self, and the surrounding world; thus, for the chronically ill, the reconstruction of one's own life story is of central importance in order to give voice to this experience.

The study of illness narratives and metaphor is also important for understanding how individuals construct meaning through illness. Lakoff and Johnson provide insight into how attending to illness narratives and metaphor is important for healthcare providers to have a deeper understanding of how individuals construct meaning around their illness. They argue that the metaphor is not primarily a matter of language, but of thought and action. “Metaphor is principally a way of conceiving of one thing in terms of another, and its primary function is for communicating a greater understanding” (pg. 36) [33]. 

## 3. Materials and Methods 

 A narrative analysis focuses on the development of a detailed plotline and characters and investigates the complexities of a situation and/or setting from the informant's point of view. Furthermore, it pays analytic attention to the following questions: (1) how did the facts get assembled; (2) for whom was the story constructed; (3) how was it made, and for what purpose; (4) what social and cultural discourses does it draw on or take for granted; and (5) what does it accomplish? Therefore, a narrative analysis is a systematic process examining *what *and *how* participants describe their social reality drawing upon the taken-for granted discourses and values circulating in a particular culture [34]. 

### 3.1. Design

 The research design for the secondary qualitative data analysis is a longitudinal narrative analysis designed to study one group of lung transplant candidates over five time points during a six-month period. Narrative is a way to represent social realities that focus on meanings and the social-cultural positions from the tellers' point of view [35]. One of the most fundamental factors of a narrative—as well as its greatest strength—is insight into how an individual “organizes their understanding of time” (pg. 3) [36]. Human beings tell narratives to give meaning to their everyday lives. Therefore, narrative inquiry is appropriate in the study of both the illness and disease narratives and the social and cultural positions reflected in and through the narrative [37]. 

### 3.2. Data Collection

The interviews were conducted across five time points three weeks apart. The intention of the interviews was to have the participants identify a symptom they wanted to focus on to manage (e.g., coughing, weakness, and fatigue). The nurse interventionist (first author) would work from the participant's representation of their target system they wished to focus on and work together with the participant to work on a plan towards strategies to manage their symptoms. Each interview lasted approximately 45 to 60 minutes. The focus of the narrative analysis for this secondary analysis was the conversations transcribed verbatim from the interview data that focused on the stories of waiting for a transplant. 

### 3.3. Sample

A purposive sample was used to investigate the illness narratives of lung transplant candidates. This secondary data set from the parent study is full of rich descriptions of illness narratives across time due to the nature of the design of the parent study. Ten participants were recruited and eight completed the study. Seven of the eight participants consented to have their interviews audio tape-recorded. The seven participants' interviews were used for this secondary data analysis. This longitudinal, repetitive design allowed us an opportunity to follow the illness narratives of the participants over time and examine how their stories of waiting were composed in the conversation during the interviews.

### 3.4. Analysis

IRB approval was granted for this secondary data analysis. A multistaged narrative analysis approach was used to analyze the illness narratives. In the preliminary phase, interviews were transcribed verbatim by a transcriptionist from the parent study who was IRB-reviewed and approved. Preproofing of all transcribed data was conducted by listening to the audiotapes while reading the transcribed interviews to check for missed words and spellings and to assure that what was said was written verbatim. Rigor was used when proof-checking the transcripts, to ensure that the context of the conversation was properly captured. To protect the anonymity of participants and practitioners, their names were changed in this report, and any identifying factors were removed or changed.

Labov and Waletzky's structural narrative analysis was an appropriate analytic strategy to analyze the narratives of waiting because it is a systematic way of analyzing stories, using the identification of beginnings, middles, and ends that occur in everyday conversation [38]. Structural elements of the story were identified: abstract, orientation, complication, evaluation, resolution, and coda [38]. In the structural analytic process 212 stories of illness narratives were coded. The 212 stories became the unit of analysis for the research question, exploring the illness narratives of lung transplant candidates. Stories were categorized into two types: (1) full-length stories (stories with a beginning, middle, and end) (*N* = 52) (2) and nonstoried events (events with an action verb and fragmented stories) (*N* = 160). From the 212 stories, within-case and across-case analyses were conducted [39]. A time ordered matrix table was then created, made from patterns of themes that were identified across all the participants over the repeated interviews (pg. 119) [40]. Only stories of waiting were identified as the focus for this secondary analysis of exploring illness narratives. Hall and Stevens, *Rigor in Feminist Research, *was adhered to in order to verify the scientific adequacy of this study. During the process of analysis, the following dimensions of rigor were adhered to: credibility, reflexivity, coherence, complexity, honesty and mutuality, naming, and dependability [41].

## 4. Results 

The findings are presented as seven waiting narratives of how waiting for a donor lung has impacted the individuals' lives.

### 4.1. Jack's Story: Waiting, Life on Hold

Jack had been on the transplant list approximately two years before the changes to the lung allocation scoring system.

*I guess most of it is my relationship with my fiancé, but a lot of it is stressful for me to always be waiting and waiting and waiting. But, the thing that is the worst is … when you put your life on hold, you put everything on hold. We wanted to get married. So, we planned on getting married … and this is something that it is just constantly … we want to get married and move on with our lives to some extent, rather than hanging in limbo, waiting forever.*

*The reason we don't get married at this point, is insurance would be a problem, if, we are getting married, I would be on her insurance policy, and that would not cover nearly as much the way I have myself set up with insurance right now. So that would be financial distress. Secondly, I'm realistic enough to know a lung transplant is not getting your appendix out, or sliver out of your finger, it's very serious and if I would die after we were married, there is a possibility that she could be stuck with bills for me, and I don't want that, and that she agrees, she agrees too, that she does not want to pay off, especially if I am gone you know. So, we decide we have to await for the transplant to get married. So, that is just eating away at us.*

Jack describes “*waiting is eating away at us,”* straining their relationship as they both hang in limbo. He describes how their stress built up to the point where his fiancé was going to break up with him.

*There was a time when it got really bad, and uh she was maybe talking about that I should move out and that got really scary and that was when I went and talked with [Healthcare Provider] and we, you know, talked. I said, “hey, what's going on? Will I ever get my lungs ever? You are ruining my life you know. I guess that's the biggest wait is the marriage, but everything. We'd like to do stuff. I can't do stuff you know, I can't do stuff anymore. We used to … Every couple of months or four times a year maybe, go and get a hotel room and relax and just let the world go by and not, not pay attention and have a nice three day weekend. We don't do that anymore. We don't, she goes places, I tell her to go by herself and she will travel to visit family. I don't want to be two and a half hours away from the hospital when they call me. I want to be here. *

Then, Jack tells the story of how he is unable to work to bring money to his household, which is also straining his relationship.

*Of course I wish I could have a job so that I could bring some money into this household and that does not happen … and that is part of all of this … you know … it kind of all blends together into we're waiting and waiting and waiting.*

Jack describes how he was affected by the new lung allocation scoring system. Jack was listed before the lung allocation scoring was implemented, which at the time meant that his time accrued while on the waiting list counted towards how close he would be in getting his donor lung. The average waiting time was two years [3]. But since the new scoring changes, Jack finds out that he (i.e., his time on the waiting list) is no longer a priority and candidates with higher severity of illness scores will have precedence for donor lungs.

*And it ah, the funny thing is when I got on the transplant list, at that time, most everyone who got on the list was transplanted within two years. So I got on the list, and then just about when my two years was coming up, they changed the way they allocated lungs and now it is the sickest person who gets them. So, the sickest person who qualifies for all of the organ tissue, the size, the blood type, all of that stuff but they have a list of who is the sickest, and I am not terrible sick, but I am not happy. (Laughs) This is not, you know, I am surviving yes, I am, but, so then they tell me, we changed now, you're not getting lungs in two years, which is a big let down, to my fiancé and I, and you know, so now it is over three years. *

Jack described how his healthcare providers continued to give encouraging news, to reassure him that he is well placed on the list. This encouragement, however, did not help how he felt.

*My last clinical visit was encouraging you know, but they're always encouraging you know. They want you to keep a positive attitude and I understand. I mean I am not dumb. (Laughs) But, I just don't see that things are going to come to an end yet, I don't see that light at the end of the tunnel, and no one can tell me. They can reassure me, and say things like, “you're well placed on the list,” which doesn't tell me anything, it just, they are saying, Well, you're up there. You know, and that it's, just hard, to still not, can't see the end of the tunnel at all you know. I, I, if I can survive like this for another five years I still might be waiting you know. I just, I don't know. This isn't really the way to live though. *

The sequence of events through Jack's story: (1) life on hold waiting to get married after transplant; (2) the financial necessity of needing to wait to get married until after the transplant; (3) his fiancé moving forward as he chooses to stay near the phone and hospital; and (4) the impact of the lung allocation scoring system on his hopes and plans. All of these events blended together in the telling of his embodied experience, and all constitute the cause for his emotional distress in his life. We gain insight into why Jack feels like he cannot see the light at the end of the tunnel. The metaphor of seeking the light at the end of the tunnel lends itself to an image of how Jack lives his experience of the emotion of waiting and how the lung allocation scoring system made an impact on his life. 

### 4.2. Edith's Story: “Listed at the Wrong Time”

Edith, has been on the waiting list approximately two years since the lung allocation scoring system was implemented. Edith tells a story of the time she was told she was no longer a priority to be transplanted due to the lung allocation scoring system rules. At the time of the interview, her present scores were not high enough for her to be considered a priority to be transplanted.

*They changed to the lung allocation program so then I got kicked right back to like, you know your time doesn't matter anymore, so it was, it was a good thing for the really sick people but a bad thing for people like myself. Cause I would have had it by now if they had not changed the rules.*

*For me personally, I was listed at the wrong time. I got listed in, in August; the following May they changed all the rules. So when I was up there in June, Dr. [Name] says to me, “Well, don't count on a transplant anytime soon.” Well, not only did that make me angry, the way he said it; and then I got to thinking, you know? “What the hell am I doing?” You know? I get, I get to see my granddaughter in [City] once a year. And there's no reason I can't fly down there and spend more time with them. You know? I, I, I'm far from getting a transplant, and, and … to ME, now this is just ME. Because of my age, and, you know? This is just a reality check. I think is what happened to me here a couple weeks ago. Um, but the reality check: I'm in my mid-sixities, the chances of my getting a lung are probably a couple years down the road, at least … unless all of a sudden I take a terrible turn for the bad, which doesn't normally happen with people in my condition.*

*The chances of me ever getting a lung transplant, at this point, I look at it as not happening. *

Edith came to terms with accepting the fact that her lung allocation scores do not list her as a priority until her condition worsens that her time accrued on the list no longer mattered.

*Let me put it to you this way: the worst part, the ABSOLUTE worst part is waiting. *

* I think helping people find … ways to occupy their time; um, for me, I have been doing this for 3 years, and I am so sick of waiting, that I am at the point where I am just going about my, life and I'm going to do what I can do when I can do it.*

*The first year … every time the phone rings you come out of your skin … And then after that, you go, “Oh God, it's not it.” And, you just, you just get so … Depression is a VERY BAD ENEMY in the waiting game, I think. And, cause I, I mean I've never been on the antidepressants in my life until I … got here.*

### 4.3. Ella's Story: “Packed Bags for Two Years”

Ella had been on the transplant list approximately two years at the time she participated in the parent study. Ella describes the day that she was listed on the transplant list and was encouraged to pack her bags because the call for a transplant could come at any time.

*Cause that's when the things started, you know? Everything starts to like, Okay, when are they going to call me, and … I'm sick of waiting, and “ya-da, ya-da, ya-da.” So then that's when … it starts to bother more so. You know? Because like when they said to me, when I first was on the list, “go home and pack your bag.” Okay, this bag is still sitting over there 2 years later; I'm going “Okay, it's probably dry-rotted by now.” *

Ella describes her emotional experience of waiting as her “pity party moods.”

*I call it my pity party mood, but it's, it's actually your state of depression. You know, waiting for something, waiting for the unknown is so scary. I always tell my sister, always every morning, I can't wait until this is over with. So I can sit and talk, and, you know and not get short of breath, and we can do this, and she just says to me, oh, it will come. *

*You know, she says, don't give up, and I says no I am not giving up, I am about to give out. You know, you just get so tired of just waiting, and waiting and waiting, your life is like totally on hold. You can't do anything, you can't go anywhere, and that is so depressing. *

Ella's illness narratives give insight into how depression sets in because of life being on hold, not being able to go anywhere or do anything due to her chronic condition. She gives us insight into courage of not giving up as a lung transplant candidate, but how the emotional toll of waiting can trample the human spirit. Since the changes to the lung allocation scores, Ella describes how she was told she was sick but not sick enough to be a priority to be transplanted. This is another example of how the disease narrative (the lung allocation scores) can over shadow the voice of the illness narratives, the human toll of waiting. The scores are not high enough to be listed as a priority, but the emotional narrative does not get acknowledged.

*It's just hard. You just, sometimes you just get to the point where you just want to say … “Take me off the list and leave me be.” You know? But then you know it's going to get worse, and worse. And then they tell you, well, see? In my … in my case, they told me at the last visit: Well—You're sick, but you're not sick and your numbers aren't high enough to be … you know? You're not as sick as you think you are.*

### 4.4. George's Story: “You're Punishing Me for Trying Hard”

George had been on the transplant list approximately one year at the time of the study. George's illness narratives show how stories can help lung transplant candidates make meaning of the past, the present, and their hope for their future.

*I finally accepted after two to three years that I had this COPD, and by the time that I accepted that I had it, I'm 5′7′′ and I weighed a 195 lbs. I'd ballooned up, and I was oxygen 24-7. I was in my room. People were bringing me food. I wasn't moving, no exercise. I was just a, I had given up and said okay this is it, I'm dying. This is the end of these, and I ain't going to try, and, ah. My son and daughter-in-law had a baby boy, grandson, and my hose for my oxygen machine would reach out on the deck, and I would sit out there on the deck, and he would be on a little seat on the table and I looked at that little guy and I thought, you know, I'm not gonna be here when he gets a little older you know. And I thought, okay, I'm gonna, I'm gonna really try to be there for him so he'll remember who I am. *

George tells a story of how he changed his life to look towards the future so that he can be around for his grandchildren. His waiting story surfaced in his interviews when he described how hard he was working to stay in shape to be a candidate for a lung transplant; however the consequence of his improved condition was that his lung allocations scores were lowered.

*Waiting, that's a little bit of a sore spot with me. Um, and I've talked to them over there about that. I mean, I'm, I'm not a complainer, but I told them you know? When I was not trying … and my FEV-1 was 17, and I didn't exercise and I was in bad shape … not taking care of myself they had me over here every other week, or once a month trying to give me a lung. And so I get in shape…and you move me down there to the 40s on the list and never gave me a call. I said “You know? You're punishing me … for … trying hard and getting in shape; but it's a double-edged sword … basically, you know?” So, it's just one of those things, but, I guess, that, what I, what I always have stick in the back of my mind and I keep telling myself, is that “If I can hang in there for another year or two, without getting that transplant, and, and maintain the way I am right now, with a certain amount of quality of life.” *

### 4.5. Clive's Story: “Pulled off the Waiting List”

Clive has been on the transplant list approximately one year since the changes to the lung allocation scoring system. Clive recalled an incident of the time they pulled him off the transplant list until his weight returned below the parameters for his body mass index. It turned out that this incident was a mistake and that Clive's weight was within the normal range for his particular case.

*I'm still a little sore with the coordinators and the stuff down there and … It hurt me pretty bad last week. They called me last Tuesday and told me they got the results from the rehab and I was at exactly uh, 250 pounds, and they were pulling me off the transplant list until I went back down to 200 or, I had to go below 230. *

*And I said “Wait a minute. I was told by the dietician, the surgeon, AND the doctor, they wanted me between 240 and 250. And I had volunteered, cause I lost 100 pounds already. I wanted to go down to 225 … and they told me “No”; with my body cavity, they wanted to keep me ABOVE 240. Now you're saying I'm off the list cause I have to go down below, uh, 230?” And she said, the coordinator said to me, she said “Well, maybe I'll check on it;” she says, “but they pulled me off the list.” I sat here for 3 hours, stewing because I do everything they tell; even going to rehab, paying for it myself. She called me back and she said “Oh, we made a mistake; you, they let you have a special situation.” And I says, “What?” I said “Do you want me to LOSE it or DON'T you?” *

* “No, they don't.” Then next she said, “We apologize for making a mistake.” And they kind of “blew it off”. I say, after all the work I do, and I keep in shape; I do everything; and, and then I said, I asked him, I said “Well wasn't the doctors, and the dieticians, all at the meeting … to pull me off the list?” And she says “Yeah, but nobody checked their records.” I lost a lot of confidence in them. “Oh, we made a mistake.” And I said “How can you make a mistake with somebody's life?”*

In Clive's story, it turned out to be a terrible mistake, but it still indicates the hurt and confusion he felt because he was told the wrong information even after following the healthcare provider's instructions. Illness narratives of the emotional toll of getting pulled off the list can get taken for granted in a busy lung transplant culture where the disease narrative dominates, such as the lung allocation scores become the priority and the emotional narrative of waiting can get dismissed. Clive's story provided insight into how much lung transplant candidates prepare their lives for this transplant and build trust with their healthcare providers. This story also provided an example of how they hang on to every word of their healthcare providers' recommendations for following the correct protocol and make changes in their lives.

*What … what hurt me the most was … I did everything that they ever asked me. I did MORE than they asked me. I did right to the letter exactly what they said. And then they call me up and tell me I did wrong. And that … that hurt; and then to wait, you know, 2- to-3 hours before they call you back. And that blew me up.*

During the three hours that he waited for the callback, Clive reflected on all he had done to lose the weight and prepare for the transplant. Clive describes his unraveling confidence and growing distrust of his healthcare team because of being told one thing and shortly thereafter being told something different. 

Illness narratives are the narratives often neglected in the face of the disease narrative. In this case, the disease narrative consisted of Clive's weight scores and the weight parameters he needed to stay within to remain on the lung transplant waiting list. The personal story gets taken for granted. Clive's case enlightens how much lung transplant candidates trust and depend on their transplant team and following their instructions. It is important for healthcare providers to not take for granted what seems like routine procedures. For example, reporting on a lung transplant candidate's status is extremely important, and getting adverse news can be experienced as a devastating blow to the candidate. Waiting for a transplant is an emotional roller coaster ride with many highs and lows, and lung transplant candidates have to work hard on a daily basis to manage their emotions. Emotional support is needed to assist them through bad news. 

### 4.6. Maeve's Story: “I'm Just Not Going to Sit Here by the Phone”

Maeve was listed approximately two years at the time of the study. Maeve describes how she has always surrounded herself with people she loves and the people who make her feel good about herself. She put her trust and faith in God's hands in dealing with the emotional toll of waiting.

* Sometimes I think “Oh God I wish I could have it tomorrow.” And then sometimes I think “Well, maybe I'm better right now, than I might be after I have it.” You know? I, a lot [sic] of things still go through my mind. But, I, I'll, I'm going to be positive. I just keep thinking “Well, when I have it, what happens; it's in, it's in God's hands.” You know? That's, that's right. You know? I, I'm just not going to sit here by the phone waiting for the phone to ring. You know? We cannot do that either. Cause I think you would go “batty” then, for sure. And I got … I got kids and grandchildren around that … you know, keep me occupied, or … keep me a-going too, so. And I think that helps a lot. I feel sorry for anybody that does not.*

Maeve's story gives insight into what it takes to be able to “let go of living by the phone.” Maeve continues on with her life, spending her day surrounded by her family and grandchildren who “keep me a-going.” Being surrounded by networks of meaningful social support has helped Maeve move forward in her life. In contrast to Edith, who was living apart from her closest emotional support network, Maeve was surrounded by her family. These stories provide a rich understanding of the kinds of emotional support and connections needed to help lung transplant candidates through these emotionally turbulent times of waiting. 

### 4.7. Meg's Story: “Seeking Support Groups before Transplant”

Meg was listed on the transplant list approximately two years during the time of the study. She has been on the transplant list two years during the time of this study. Meg describes how she seeks conversation with other lung transplant candidates who are going through similar life situations.

*Through my volunteering I have met people that have gotten transplants because of certain things. I haven't met a lot of people that's gotten lung transplants though, but I have met people that had lung problems, kidneys and hearts and stuff like that. After talking with them … I kind of get a clearer picture of what my life can be like AFTER transplant.*

* It's uh support groups BEFORE that I'm, I'm more interested in, and there's not a lot around. And I actually did meet a, a fellow a couple weeks ago, that um, would like to start off something like that, and he asked me if I would help out. And I thought “Well, yeah, I would love to,” because I would love to meet with people that um, prior to transplant. *

*And it's … it's ODD for me when, when I volunteer, I'm usually um, one of the people that … you know, are sick, you know, pre-transplant. There's not a lot of people uh, you know, volunteering that are pre-transplant. *

Meg's illness narratives help us to understand how valuable being part of a community is where she can be amongst other lung transplant candidates who are experiencing similar issues of pretransplant life. We learn that she desires human connection knowing that she is not alone during this time of waiting. 

## 5. Conclusion and Discussion 

The findings of this structural narrative analysis provide insight into the context of situational events that cause the roller coaster of emotional distress in patients' lives. Labov's and Waletzky structural analysis focuses on the event, and therefore, by using it, we gain insights into various situational events of waiting for transplant [38]. Participants from this secondary analysis were affected by the lung allocation scoring system and described how their personal lives have been affected by no longer being a priority to be transplanted. We gain insight into the lung transplant candidates' understanding of time and the urgency for their personal stories of suffering to be heard.

Jack tells his story of how his marriage to his fiancé is on hold. He described how his relationship is strained because of all the things he cannot do with his fiancé, such as travel with her because he fears he might miss the “transplant” call, insurance concerns, and his inability to work due to his chronic condition. Jack states “We want to get married and move on with our lives.” He gives us insight into how time of waiting, “is just eating away at us” and the urgency for his emotional pain of his relationship to be heard. 

 Edith described how waiting caused her depression, and how the new lung allocation system and her getting older will decrease her chances of getting a transplant. For Edith, we gain insight into how she is at a point in her life where she is going forward: “I'm going to do what I can do, when I can do it.” She has come to a place in time where she is going to take charge of her life and no longer wait by the phone. 

 Ella's story provided insight about how the lung allocation scores privilege the physiological narrative, such as “you're sick but you're not sick enough,” which overshadows her emotional narrative of how waiting takes a toll on the human spirit. The urgency of Ella's illness narrative is “I am not giving up, I am about to give out.” Ella's story raises the questions of how can healthcare providers enhance attending to the emotional exhaustion of waiting. 

George shares the time in his life that was a turning point when he decided he is going to be there for his grandson. The urgency of his illness narrative is how he feels punished because he is working hard to maintain his status as a lung transplant candidate, but now his improved condition decreased his lung allocation score.

Clive's story provided an example of how much trust patients have in their healthcare providers, and the emotional impact of being told they are being taken off the transplant list. In Clive's narrative, he gives insight into how much patients surrender to what their healthcare providers ask of them and more. In Clive's case he was pulled off the transplant list because of a mistake in interpreting his weight. The urgency of Clive's story is a reminder for healthcare providers to understand how extremely important communication between the health caregiver and candidate is to promote trust.

Maeve tells the story of how she resists feeling confined waiting by the telephone, which she attributes to being surrounded by her family and friends and her faith in God. Maeve's story provides a glimpse of the importance of the time waiting for a transplant to be amongst people who give meaning to her life and not allowing the wait for the “transplant” phone call to consume her life. 

Meg's story gave insight into the importance of emotionally connecting to others who are experiencing similar experiences before transplant. Meg gives us insight into the importance of this time to be amongst others experiencing waiting for transplant, rather than emphasizing the destination of life after transplant. 

The waiting narratives of the seven lung transplant candidates since the implementation of the lung allocation system provided (1) insight into the lung transplant candidate's personal biography as it was disrupted by waiting; (2) information that they wanted their healthcare providers to understand about how waiting was affecting their personal and emotional lives; (3) revelations about how these participants felt about the new lung allocation scoring system resulting in their time accrued on the waiting list no longer counting and resulting in their “new” scores being not high enough for them to be considered a priority; (4) waiting by the phone consumed and confined them from being able to live their lives; (5) lung transplant candidates have built great trust in their healthcare providers; and (6) their need for emotional support from other pretransplant candidates. 

Reflecting on the narrative questions: (1) for whom was the story constructed, (2) how was the story made, and (3) for what purpose, the authors came to the following conclusions. 

The stories were primarily constructed for the lung transplant candidates themselves who gave voice to the suffering of waiting. By the act of “telling” their story they were making sense of their lives since the lung allocation scoring system was implemented. The act of telling their story was a place for them to give voice to their lived experience of waiting. Stories were made because the interviewers enabled their stories of waiting to be told by creating the environment that invited their stories and allowing them to tell the stories the way they needed to tell them. The purpose of their story was to give voice to the personal sacrifice of being a lung transplant candidate and how the lung allocation scoring system impacted their lives. 

Overall, candidates did not see the interviewers as representing the lung allocation system. The interview was a safe place, a place where they believed they could contribute and help other lung transplant candidates experiencing similar circumstances. They wanted their stories of waiting to be heard, and this interview was a place for them to tell their story. 

In summary, lung transplant candidates' stories provide a window into how each of them is personally impacted by the waiting process. The narratives can also help healthcare providers understand important aspects of an individual's life that are so often overlooked by the discourse of the routine systematic screenings during check-ups. It is important for lung transplant candidates to have a place to share their illness narratives of waiting for a transplant and to have their experiences acknowledged, valued, and validated by their healthcare providers.

This study extends Macdonald's study by conducting a structural narrative analysis of their stories over a longitudinal period of time [21]. This study extends Naef and Bournes' study by exploring the experiences of waiting after the changes in the lung allocation scoring system were implemented [22]. This study also extends the work of Yorke and Cameron-Traub who analyzed patients' perceived care needs [23]. This study focused on empowering patients' voices and identified how healthcare providers can constrain illness narratives by following routine protocols. Therefore, this study explicates communication possibilities and identifies areas for future research that could explore how healthcare providers can enhance their skills necessary to attend to patients' stories. 

This study concurs with the results of the research conducted by Festle [42]. Festle's work provides us with a rich and deeper understanding of lung transplant candidates' narratives of waiting and coping. This secondary analysis extends Festle's work by exploring the lung transplant candidates who were impacted by the lung allocation scoring system implemented in 2005. Exploring the illness narratives of lung transplant candidates identified the need for future research to address the emotional reactions of waiting for a transplant. 

Research is lacking in best practices for healthcare providers to educate lung transplant candidates about the stresses of waiting for transplant and the importance for them to talk about it, bring up their concerns and become proactive if depression and anxiety start to impact their life. More research should explore barriers that impede lung transplant candidates from seeking help for depression, anxiety, and emotional distress. 

### 5.1. Recommendations

 Healthcare providers should create encounters that enable lung transplant candidates to tell their stories from their points of view. Healthcare providers can enact self-reflective practices by being aware of their own explanatory models. By being aware of our own biases and agendas, we can help bridge communication, to see what we take for granted in understanding the patient's explanatory model. Healthcare providers should learn the necessary narrative skills to be able to listen attentively to complicated narratives, such as narratives of the suffering of waiting. Charon, a pioneer in introducing narrative medicine, stated that narrative skills can be used to enhance how healthcare providers attend to and listen to representations of patient's stories [43–45]. More research is needed to explore the process of dialogue and empowering patients' voices in transplant populations. Research using structural narrative analysis methods should be further explored to examine conversations between healthcare providers and patients to enhance skills of communication. Healthcare providers caring for transplant candidates could benefit from learning skills of narrative medicine to enhance how they listen and attend to the human condition and suffering from waiting for the call for transplant.
